# Predictors of mortality in heart failure patients with reduced or mildly reduced Ejection Fraction: The CASABLANCA HF Study

**DOI:** 10.1186/s43044-024-00436-y

**Published:** 2024-01-22

**Authors:** Abdessamad Couissi, Meryem Haboub, Siyam Hamady, Taha Ettachfini, Rachida Habbal

**Affiliations:** 1 Cardiology Department of Ibn Rochd University Hospital , Casablanca, Morocco; 2 Hassan II University, Faculty of Medicine and Pharmacy of Casablanca, Casablanca, Morocco

**Keywords:** Heart failure, Patient readmission, Mortality, Prognosis, Morocco

## Abstract

**Background:**

Heart failure (HF) poses a significant public health challenge throughout the world and Morocco. Our objective was to delineate the epidemiological characteristics of Moroccan patients living with chronic heart failure and to identify prognostic factors correlated with CHF mortality.

**Results:**

A total of 1344 patients participated in this study, with survival rates at 3, 6, and 10 years recorded at 75.2%, 60%, and 34%, respectively. During the follow-up, 886 patients succumbed, representing a mortality rate of 65.9%. A Cox regression model, utilizing baseline candidate variables, was developed to predict cardiovascular (CV) mortality. Predictors all of which had a *P* value less than 0.05 included age, left ventricular ejection fraction (EF) at commencement (< 35%), hypertension, male sex, anemia, creatinine levels, and the number of hospitalizations due to HF decompensation. Notably, the frequency of hospitalizations emerged as the most potent predictor of mortality, with an HR of 2.5 (95% CI [2–2.9]). Almost 90% of patients with three or more readmissions for HF decompensation experienced mortality by the follow-up’s conclusion.

**Conclusions:**

This study offers valuable insights into risk factors and clinical outcomes in HF patients in Morocco. Factors such as male gender, advanced age, a history of hypertension, lower systolic blood pressure, rehospitalizations for HF decompensation, low left ventricular ejection fraction, anemia, and elevated creatinine levels were associated with increased mortality. Medical and health services managers should be aware of the substantial burden and future challenges posed by HF in Morocco, prompting the adoption of multidisciplinary strategies for its management and care.

## Background

The historical roots of heart failure extend to antiquity, with symptoms purportedly described over 3500 years ago in the remains discovered within a tomb in the Valley of the Queens in Egypt [1]. Since that time, heart failure has posed a substantial and enduring challenge to the field of medicine. Despite advancements in science, technology, and an enhanced understanding of heart failure physiopathology, it remains a substantial challenge to health care professionals.

The American Heart Association defines Heart Failure (HF) as a complex clinical syndrome that results from a functional or structural heart anomaly impairing ventricular filling or ejection of blood to the systemic circulation [2].

Heart failure is a global public health concern affecting millions of people and posing an important social and economic burden more pronounced in developing countries [3]. Morocco is an emerging country experiencing an important transformation in the composition and habits of its society. In fact, like North African countries its population adopted a Western lifestyle in the last decades [4] which has led to an increase in the incidence of Diabetes, Hypertension, obesity, and other Cardiovascular risk factors; Causing an increase in cardiovascular disease and heart failure incidence. Nearly one million Moroccans are living with HF and this number will increase in the future years [5].

The World Heart Federation claims in its annual report of 2023 that cardiovascular diseases (CVDs) are the leading cause of death globally, taking an estimated 20.3 million lives in 2021. Lindstrom et al. [6] the same report predicted that one-third of cardiovascular deaths can be prevented by improving cardiovascular Health, especially in developing countries. Some studies have also reported a great disparity between HF mortality and prognosis between low- and middle-income countries and high-income countries with some authors stating that the lack of adherence to optimal medical-guided therapy may have a key role in these disparities [7, 8].

Prior investigations into heart failure in Morocco have predominantly concentrated on the epidemiological dimensions of the condition, with limited information pertaining to prognosis and predictors of mortality in this geographical context.

The present study endeavors to contribute a more thorough understanding of the burden imposed by heart failure in Morocco, aiming to elucidate the factors that influence its outcomes within population and to facilitate the provision of more efficient and targeted health services tailored to the specific needs of the Moroccan community.

## Methods

We conducted a retrospective observational investigation that enrolled patients diagnosed with heart failure with reduced ejection fraction (HFrEF) or heart failure with mildly reduced ejection fraction (HFmEF). The study was conducted within the Cardiology department of Ibn Rochd Hospital in Casablanca, spanning from January 2012 to December 2021, with subsequent follow-up until March 2023. Data were systematically gathered from the Heart Failure Registry, and both mortality and incidents of HF decompensation were obtained through direct communication with patients and their families via telephone.

Diagnostic assessments were carried out using Vivid 7 (General Electrics) echocardiography, with left ventricular ejection fraction (LVEF) estimated using Simpson’s method. Inclusion criteria comprised patients exhibiting reduced or mildly reduced ejection fraction, while those with missing data and individuals with LVEF exceeding 49% were excluded.

For statistical analyses, continuous data are expressed using means and standard deviations (SD). Categorical variables were expressed as percentages and subjected to the chi-square test for comparison. Given that the primary endpoint was mortality, survival over time was calculated using the Kaplan–Meier method. Differences in mortality between subgroups were assessed using the log-rank test. Predictors of all-cause mortality were identified through a univariate Cox proportional hazard regression model. All statistical analyses were performed using IBM SPSS Statistics version 23.

## Results

### Patient characteristics

The study commenced with the inclusion of a total of 1546 patients, from which 202 individuals were subsequently excluded due to insufficient data, resulting in a final cohort of 1344 patients over a ten-year follow-up period. A comprehensive summary of pertinent patient information is presented in Table 1.

**Table 1:** Characteristics of the studied population

	Survivors (*N* = 458)	All causes deaths (*N* = 886)	*P* value
Age (y)	59.2 (12.2)	64 (13.5)	< 0.001
Gender (M), *n* (%)	264 (57)	587 (66)	0.04
SBP (mmHG)	128.2 (22)	117 (21)	< 0.001
DBP (mmHG)	74( 12)	69 (11.5)	< 0.001
HR (bpm)	78( 17)	85.9 (17)	< 0.001
HF etiology *n* (%)			
Ischemic heart disease	342 (74.6)	734 (82)	0.3
Valvular heart disease	41 (8.9)	94 (10.6)	
others	69 (15)	58 (6.5)	
Rehospitalizations rate			
0–1	386 (84)	633 (71)	
2 or more	72 (15)	253 (28.5)	< 0.001
NYHA CLASS *n* (%)			
I–II	404 (88)	730 (82)	
III	48 (10)	144 (16)	
IV	6 (1)	10 (1)	0.68
Previous Stroke *n* (%)	24 (5)	32 (3)	
Previous history of AF *n* (%)	104 (22)	181 (20.2)	
Hypertension *n* (%)	190(41)	470 (53)	0.04
Diabetes *n* (%)	176(37)	362 (40)	0.21
Menopause n (%)	168 (36)	233 (26)	0.2
ESRD *n* (%)	88(19)	51 (5.7)	0.83
Hb (g/dL)	13.2(2.08)	11.5 (1.9)	< 0.001
Ht (%)	40 (20.2)	38 (16.4)	0.62
CRP (mg/L)	17 (27)	27 (30)	< 0.001
Serum creatinine (mg/dL)	10.6 (4.2)	12.39 (4.6)	< 0.001
eGFR (ml/min)	66.3 (13.2)	54.2 (10.5)	
Serum Urea (g/L)	0.48 (0.3)	0.51 (0.4)	0.32
Serum Sodium (mmol/L)	137 (3.2)	137.9 (3.3)	0.82
K (mmol/L)	4.7(0.8)	4.9 (0.9)	0.9
AST (U/L)	24 (9.2)	27 (8.8)	0.23
ALT (U/L)	18(6.6)	23 (7.5)	0.3
LVEF (%),	36 (9.4)	35 (9.8)	< 0.001
ACEI/ARB *n* (%)	379 (82.7)	681 (76.8)	0.5
Beta-blockers *n* (%)	360 (78.6)	669 (75)	0.73
MRAs *n* (%)	200 (43.66)	340 (38.3)	0.42

*AF* Atrial fibrillation, *CRP* C-reactive protein, *DBP* diastolic blood pressure, *ESRD* end-stage renal disease, *eGFR* Estimated Glomerular Filtration Rate, *HF* heart failure, *LVEF* left ventricular ejection fraction, *NHYA* New York Heart Association, *Hb* hemoglobin, *Hct* hematocrit, *SBP* systolic blood pressure

Among the included patients, 61.6% were male, and the mean observed age was 61.48 years. Ischemic heart disease was identified as the primary etiology of heart failure in 81.02% of cases. The prevalence of diabetes and hypertension within the entire patient cohort was noted at 40% and 51.4%, respectively. The median value of left ventricular ejection fraction was determined to be 36.2%.

### Clinical outcomes

At the conclusion of the follow-up period, the overall mortality rate stood at 65.92%, indicating that 886 patients within the cohort had passed away. Additionally, the survival rates at 3, 6, and 10 years were 75.2%, 60%, and 34%, respectively.

#### Prognostic predictors

Upon univariate Cox regression analysis, several factors were identified as prognostic predictors associated with a higher mortality rate. These factors include male gender, advanced age, lower systolic blood pressure, a history of hypertension, two or more rehospitalizations for heart failure (HF) decompensation, left ventricular ejection fraction (LVEF) below 35%, and elevated levels of creatinine, as detailed in Table 2.

**Table 2:** Univariate cox-proportional hazard models for all-cause mortality in the study population *N* = 1344 patients

	HR (95% CI)	*P* value
Male	1.07 (1.04–1.1)	0.04
Age (per 10 years increment)	1.3 (1.1–1.5)	< 0.001
History of Hypertension	1.2 (1.1–1.3)	0.05
Re hospitalizations rate (2 or more)	2.5 (2–2.9)	< 0.001
EF (per 5 decrease below 35)	1.09 (1.02–1.2)	< 0.001
Creatinine Levels	1.11 (1.01–1.2)	< 0.001
Systolic blood pressure	0.9 (0.80–0.98)	0.02

#### Time-to-event analysis

**Fig. 1:**
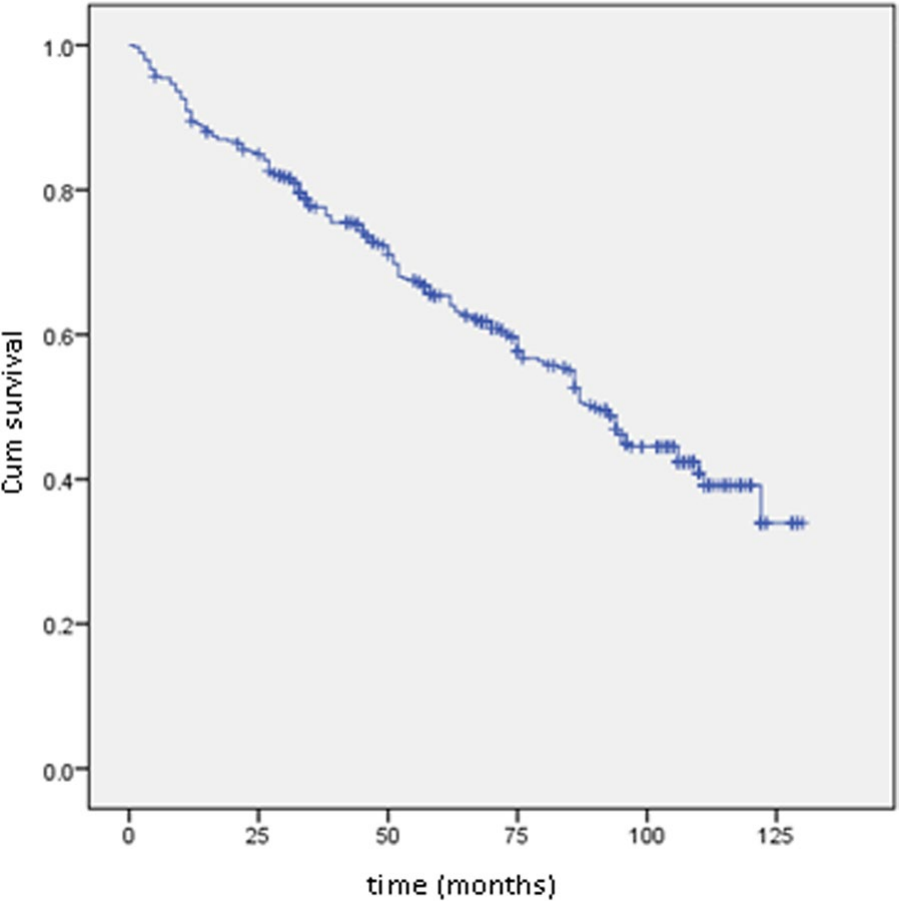
Kaplan–Meier curve showing overall mortality

**Fig. 2:**
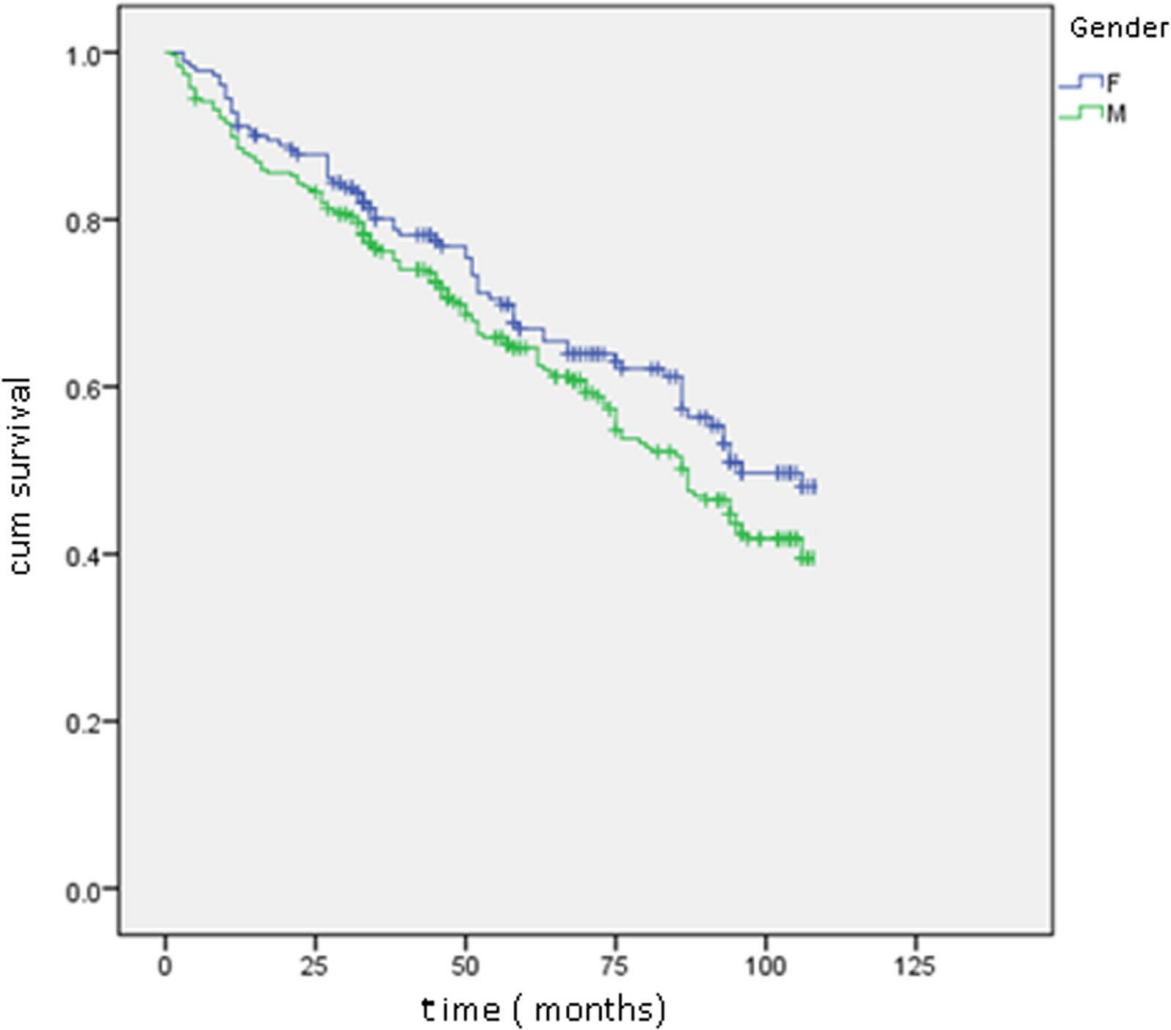
Kaplan–Meier curves: survival curves of males and females. Log rank 0.044

**Fig. 3:**
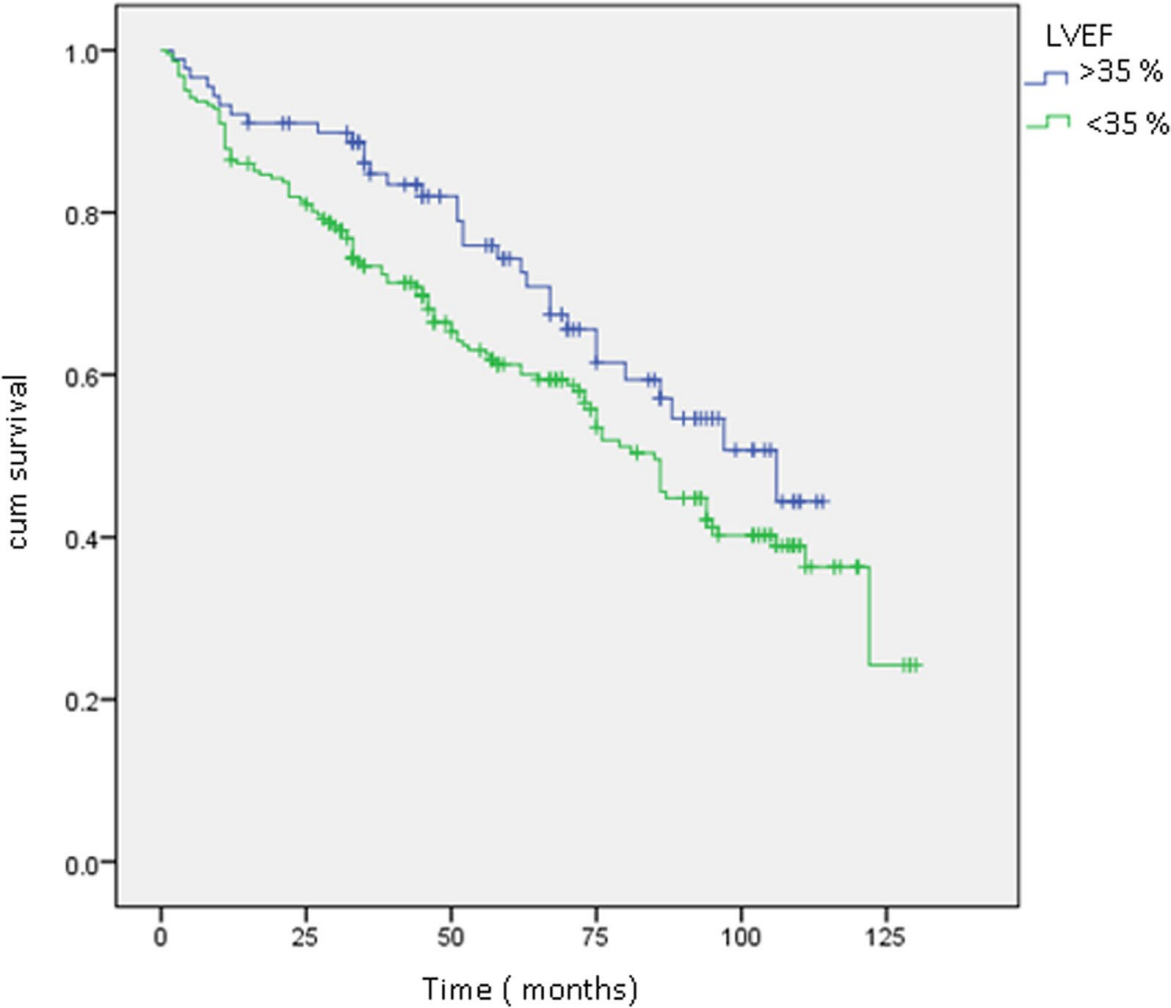
Kaplan–Meier curves: survival curves depending on LVEF. Log rank 0.02

**Fig. 4:**
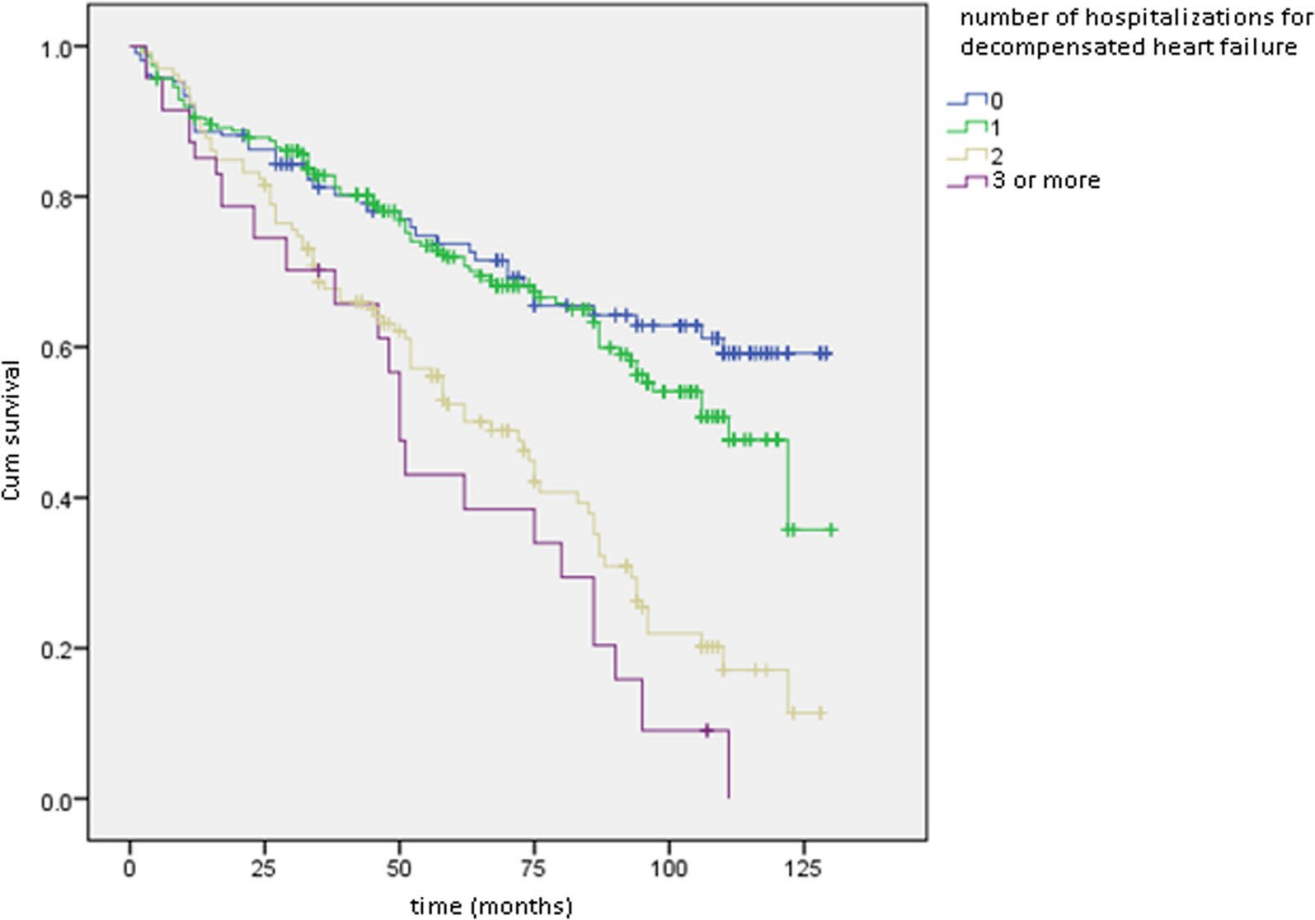
Kaplan–Meier curves: survival curves depending on number of hospitalizations. Log rank  < 0.001

The overall survival curve is depicted in Fig. 1, while Figs. 2, 3, and 4 illustrate survival curves across different subgroups. Notably, patients with two or more hospitalizations due to heart decompensation exhibited the most pronounced mortality rate, with nearly all patients experiencing three or more hospitalizations succumbing by the conclusion of the follow-up period (*P* < 0.001).

Patients with lower LVEF (< 35%) also demonstrated higher mortality, with a distinct separation of survival curves evident after 12 months (*P* < 0.001). Male patients exhibited a slightly elevated mortality rate compared to their female counterparts (*P* = 0.04). Finally, creatinine levels exhibited a proportional correlation with the mortality rate.

## Discussion

We sought to explore the aspects of chronic heart failure in the Moroccan population; the overall 10-year all-cause mortality was 65.9% with a median follow-up of 58 months. similar findings were found by A recent meta-analysis that included 60 studies mostly conducted in Europe or North America where survival rate of 1, 2, 5, and 10 years was 86.5%, 72.6%, 56.7%and 34.9% respectively [9]. Another study that enrolled participants from low-income countries concluded a significantly higher one-year mortality in Africa (30.6%), and Asia (13.1%), compared to patients from South America (8.6%) and the Middle East (9.1%) *P* < 0.001 [10]. These results may be underestimated for many reasons: HF is underdiagnosed in these countries, and guideline-directed medical therapy GDMT is not often applicable to treat patients, and poor-quality health services. The absence of Awareness and Prevention programs launched in these countries. Some studies report a twofold increase in the risk of in-hospital mortality and post-discharge events compared to high-income countries [11].

The predominance of the male population (nearly two-thirds) may be explained by tobacco being an important risk factor of ischemic heart disease (more than 80% of HF etiology in our study) predominant in the Moroccan male population. Male gender was also an independent risk factor for all-cause mortality. A study conducted in Saudi Arabia found no significant difference in survival between women and men [12]. Our findings were reported in a recent UK study in which women had a 14% age‐adjusted lower risk of all‐cause mortality [13]. However, a recent Canadian study found that women remain at higher risk than men of dying or developing heart failure in the subsequent 5 years after acute coronary syndrome [14]. These conflicting data may be due to different population characteristics and health care systems.

Age was a strong predictor of mortality, and elderly patients with CHF have a poor prognosis, particularly if their heart failure symptoms are caused by LV systolic dysfunction [15]. As living conditions improve, the elderly population in Morocco will increase significantly by 2030. The High Commission for Planning (HCP) projects a significant demographic shift in Morocco by 2030, with the senior population aged 70 and above exceeding six million, representing a substantial 42% increase from 2021. This transformative surge is anticipated to reshape the societal landscape, as seniors are expected to constitute 15.4% of the total population, as highlighted in the HCP report.

The rehospitalization rate emerged as the foremost risk factor for mortality in this study, revealing a threefold increase in overall heart failure mortality. Although this association is acknowledged in existing literature [16, 17], the resource constraints in Morocco have resulted in the reservation of heart failure hospitalization predominantly for critical cases. As illustrated in Fig. 4, a substantial proportion of patients with three or more hospitalizations experienced mortality over a 10-year follow-up. These observations underscore heart failure decompensation as a pivotal factor indicative of structural and functional cardiac anomalies.

Whether the mortality of patients with acute heart failure onset is higher than those with acute decompensation of chronic heart failure is still subject to debate with conflicting results in recent studies [18, 19] Unfortunately, we could not analyze it due to lack of data. What is sure is that lack of treatment adherence is strongly correlated to mortality and rehospitalization [20]. Our patients had a significant lack of adherence to medical therapy and only a few of them had reached the maximum dose of beta-blockers and ACE making the prevention of cardiovascular morbidity, close follow, and patient education crucial for reducing their mortality in our country.

A reduced left ventricular ejection fraction (LVEF) is indicative of the severity of infarcted zones and fibrosis, and it is consistently linked to an elevated risk of arrhythmia and sudden cardiac death, as corroborated by numerous studies [21, 22]. In our investigation, as depicted in Fig. 3, an LVEF lower than 30% exhibited a statistically significant association with mortality, as indicated by a log-rank value of 0.02. These findings underscore the prognostic relevance of LVEF in our study cohort, aligning with the established understanding of its implications for adverse cardiac outcomes.

In this study, the presence of anemia demonstrated a significant association with higher mortality, a result consistent with findings reported in numerous other studies. The relationship between anemia and mortality was proportional to the severity of the anemia many studies tried to find an explication to this: the difficulty of management of HF patients with anemia, accentuated inflammatory reaction, and nutritional deficiency of iron may all play a role in this higher mortality [23, 24]. Whether the anemia is a consequence of severe HF or a cause of poor outcomes is still an enigma [25]. Hence the benefit of blood transfusion to improve the prognosis of these patients is still a subject of debate as the transfusion therapy can lead to volume overload and ischemic events in HF patients [26]. Some studies in hemodynamically stable cardiac patients suggest reserving blood transfusion to patients with a Hb level below 80 g/L [27].

C-reactive protein is a direct marker of Inflammation which plays an important role in HF pathophysiology[28]. This study found a strong correlation between C-reactive protein and mortality, this result was also found in a Japanese study published in 2019 [29], and the mechanism in which inflammation increases mortality is not fully understood; it is believed that it may result from both a direct myocardial release in consequence of hemodynamic overload and systemic cellular production caused by reduced tissue perfusion and associated hypoxia [30]. Hence, the use of CRP as prognostic marker may be important in the stratification of mortality risk among HF patients.

Our study found that a decrease in renal function was associated with higher overall mortality, and many other studies shared the same finding [31, 32]. This was not surprising for the decrease in renal function which is a direct reflection of deteriorating organ perfusion. The major cause of renal function deterioration is either type 1 CRS (acute cardio-renal syndrome) or type 2 CRS (chronic cardio-renal syndrome). While both hypoperfusion and high glomerular pressure play a role in renal deterioration, type 2 CRS seems to have higher RAAS and SNS activation [33]. Hence, these patients benefice greatly from beta-blockers and ACE\ARBS treatment.

Blood pressure was inversely correlated with mortality; an Italian study that enrolled 6975 patients shared the same finding [34] and lower blood pressure may be a sign of lower cardiac output which is related to impaired ventricular function scaring and hence high risk of death.

Despite the robust identification of hyponatremia and diabetes as significant risk factors in prior studies, [35–37], our investigation failed to establish a significant correlation between these factors and mortality within our study cohort. It is noteworthy to acknowledge that this lack of correlation may be attributed to the inherent limitation of our study, specifically the relatively constrained size of the study population. The restricted sample size may have influenced the statistical power and, consequently, the ability to detect significant associations.

A Scottish study published in 2017 has found that HF is as “malignant” as some of the common cancers in both men and women [36, 37]. It is believed that heart failure gravity is underestimated by the general population even in rich countries [38] and that spreading awareness has an important impact on reducing cardiovascular-related morbidity and mortality [39]. This will have a greater effect in developing countries like Morocco.

Linking these insights to the context of Morocco and other developing countries, the adoption of a Mediterranean lifestyle emerges as a potential strategy for cardiovascular health as a recent British study published in 2022 found that promoting Mediterranean and low-fat diets reduced cardiovascular mortality, myocardial infarction, and the incidence of strokes as stated by [40].

The challenges faced in heart failure management in Morocco highlight areas where healthcare infrastructure and practices need serious improvement. Limited resources, access to specialized care, population aging, and gaps in patient education can hinder optimal heart failure management.

## Conclusions

This study provides valuable insights into risk factors and clinical outcomes in HF patients in Morocco, male gender, age, low systolic blood pressure, history of hypertension, rehospitalizations for HF decompensation, low LVEF anemia, and high levels of creatinine were associated with increased mortality.

The implications of these findings are substantial for medical and health services managers. The identified risk factors underscore the significant burden and forthcoming challenges posed by HF in Morocco. To address this, a strategic shift toward multidisciplinary approaches in the management and care of this disease is imperative. By understanding these factors, healthcare professionals and administrators can develop nuanced strategies tailored to the specific needs of HF patients in the Moroccan context, potentially optimizing outcomes and resource utilization.

## Limitations

This study is subject to several limitations that should be noted. First, 15% of the population had to be excluded due to missing data. Additionally, the analysis of smoking-related data was not feasible due to a lack of information, and certain variables, such as C-reactive protein, had more than 25% missing values. The study’s design also prevented us from differentiating between cardiovascular and all-cause mortality, primarily due to the absence of medical informatization in Morocco.

It is important to highlight that the sample size of 1344 patients may not be representative of the heart failure population, which is estimated to be nearly one million patients. Despite this limitation, the study offers valuable insights into the characteristics of the included cohort.

Due to the design of the study, a comprehensive reexamination of all patients for the evaluation of Guideline-Directed Medical Therapy (GDMT) was not conducted. Instead, our focus was directed solely toward the assessment of heart failure medications.

We acknowledge the need for caution in generalizing these findings, given the current limitations. We hope that a future research on a national and potentially Arabic scale, with a larger and more diverse population, would contribute to confirming our current findings and provide a more comprehensive assessment of the impact of Guideline-Directed Medical Therapy (GDMT) adherence on heart failure patients.

## Data Availability

The datasets used and/or analyzed during the current study are available from the corresponding author on reasonable request.

## Abbreviations

AF: Atrial fibrillation
CRP: C-reactive protein
DBP: Diastolic blood pressure
ESRD: End-stage renal disease
HF: Heart failure
HFrEF: Heart failure with reduced ejection fraction
HFmEF: Heart failure with mildly reduced ejection fraction
HFpEF: Heart failure with preserved ejection fraction
GDMT: Guideline-directed medical therapy
LVEF: Left ventricular ejection fraction
NHYA: New York Heart Association
Hb: Hemoglobin
Hct: Hematocrit
SBP: Systolic blood pressure
